# Time Dependency of Non-Thermal Oxygen Plasma and Ultraviolet Irradiation on Cellular Attachment and mRNA Expression of Growth Factors in Osteoblasts on Titanium and Zirconia Surfaces

**DOI:** 10.3390/ijms21228598

**Published:** 2020-11-14

**Authors:** Linna Guo, Ziang Zou, Ralf Smeets, Lan Kluwe, Philip Hartjen, Claudio Cacaci, Martin Gosau, Anders Henningsen

**Affiliations:** 1 Department of Oral and Maxillofacial Surgery, University Hospital Hamburg-Eppendorf, 20246 Hamburg, Germany; xiangya.zou@gmail.com (Z.Z.); r.smeets@uke.de (R.S.); kluwe@uke.de (L.K.); p.hartjen@uke.de (P.H.); m.gosau@uke.de (M.G.); a.henningsen@uke.de (A.H.); 2 Division Regenerative Orofacial Medicine, Department of Oral and Maxillofacial Surgery, University Hospital Hamburg-Eppendorf, 20246 Hamburg, Germany; 3 Department of Neurology, University Hospital Hamburg-Eppendorf, 20246 Hamburg, Germany; 4 Implant Competence Centrum, Weinstr. 4, 80333 Munich, Germany; dr.cacaci@icc-m.de

**Keywords:** ultraviolet light, non-thermal plasma, osteoblast-like cells, titanium, zirconia

## Abstract

Ultraviolet (UV) light and non-thermal plasma (NTP) are promising chair-side surface treatment methods to overcome the time-dependent aging of dental implant surfaces. After showing the efficiency of UV light and NTP treatment in restoring the biological activity of titanium and zirconia surfaces in vitro, the objective of this study was to define appropriate processing times for clinical use. Titanium and zirconia disks were treated by UV light and non-thermal oxygen plasma with increasing duration. Non-treated disks were set as controls. Murine osteoblast-like cells (MC3T3-E1) were seeded onto the treated or non-treated disks. After 2 and 24 h of incubation, the viability of cells on surfaces was assessed using an MTS assay. mRNA expression of vascular endothelial growth factor (VEGF) and hepatocyte growth factor (HGF) were assessed using real-time reverse transcription polymerase chain reaction analysis. Cellular morphology and attachment were observed using confocal microscopy. The viability of MC3T3-E1 was significantly increased in 12 min UV-light treated and 1 min oxygen NTP treated groups. VEGF relative expression reached the highest levels on 12 min UV-light and 1 min NTP treated surfaces of both disks. The highest levels of HGF relative expression were reached on 12 min UV light treated zirconia surfaces. However, cells on 12 and 16 min UV-light and NTP treated surfaces of both materials had a more widely spread cytoskeleton compared to control groups. Twelve min UV-light and one min non-thermal oxygen plasma treatment on titanium and zirconia may be the favored times in terms of increasing the viability, mRNA expression of growth factors and cellular attachment in MC3T3-E1 cells.

## 1. Introduction

Dental implants are a proven concept to replace missing teeth [1,2]. In order to achieve successful long-term stable dental implants, osseointegration, which is a functional and structural connection between the surface of the implant and the living bone, has to be established [3,4]. Rapid and predictable osseointegration after implant placement has been a key point of research in dental implantology. Since the efficiency of osseointegration is closely related to the implants’ surface, many modifications have been published in order to improve the biomaterial surface topography, and chemical modifications [5,6,7].

Surface modifications and treatments that enhance hydrophilicity of dental implants have been proven to promote osteo-differentiation, indicating that hydrophilic surfaces may play an important role in improving osseointegration [8]. Recent studies have reported that storage in customary packages may result in time-dependent biological aging of implant surfaces due to contamination by hydrophobic organic impurities [9,10]. Ultraviolet (UV) light and non-thermal plasma (NTP) have shown to be able to significantly increase the hydrophilicity and oxygen saturation of the surfaces by changing the surface chemistry, e.g., by increasing the amount of TiO2 induced by UV light and the amount of reactive oxygen/nitrogen species (ROS/RNS) by NTP [11,12]. Additionally, UV irradiation and NTP can be used for the decontamination of implants and biological surfaces. including the destruction of microorganisms and removing biofilms [13,14,15]. It was reported that UV light and NTP may enhance the attachment and proliferation of osteoblasts without affecting their osteogenic differentiation potential [16,17]. Supporting the initial adhesion and retention of cells, UV light and NTP can be regarded as a promising technology for dental implant surface modification.

Due to the small sizes of the required devices and possible short treatment times, UV light and NTP surface treatments could be easily integrated into the routine of a dental practice. In order to improve the use of these two surface treatment methods in clinical practice, the determination of an effective processing time would be of particular interest. Iwasa et al. found that biological aged titanium surfaces became superhydrophilic after 48 h of UV treatment, enhancing the bioactivity of these surfaces [18]. In previously published studies, titanium and zirconia surfaces were treated by UV light and NTP using exposure times of 12 min. Either method was able to significantly increase the wettability and oxygen saturation of the surfaces and decrease the carbon remnants indicating the possibility of shorter processing times for these two methods [12,19,20]. Whether the effects of UV light and NTP treatment in increasing the biological activity of implant surfaces depend on processing time is still unclear.

Due to their excellent biocompatibility and mechanical properties, titanium and zirconia are commonly used as dental implant materials. In previous studies, we revealed that 12 min non-thermal oxygen plasma and UV-light treatment significantly increased the cellular activity of human gingival fibroblasts and murine fibroblasts on titanium and zirconia surfaces that were stored for 4 weeks [21]. However, an ideal treatment time has not been determined. Therefore, the aim of this study was to investigate the effect of non-thermal oxygen plasma and UV irradiation treatment after different processing times on viability, cell attachment and mRNA expression of vascular endothelial growth factor (VEGF) and hepatocyte growth factor (HGF) in murine osteoblast-like cells.

## 2. Results

### 2.1. Viability

**Figure 1:**
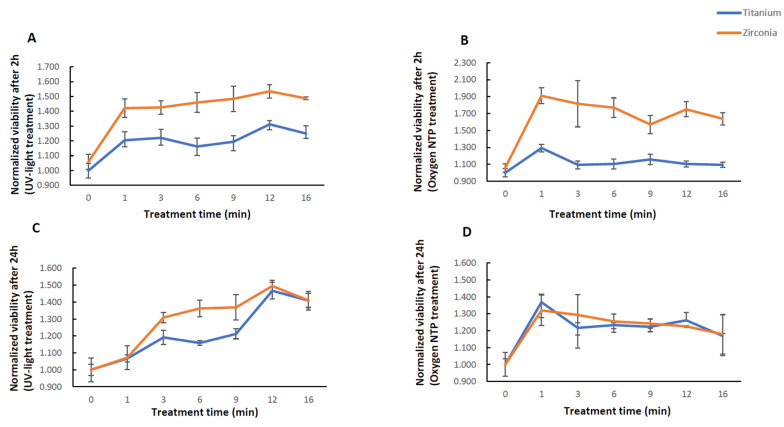
Viability of murine osteoblast-like cells (MC3T3-E1) on controls and time-course surface treated titanium and zirconia disks with UV-light and oxygen non-thermal plasma (NTP) after 2 (**A**,**B**) and 24 h (**C**,**D**) of incubation.

Generally, normalized viability values were higher on zirconia surfaces compared to titanium surfaces. However, the trend of changes in the viability of cells on titanium was similar to that on zirconia after 2 and 24 h of incubation. Viability increased with the treatment duration on UV-light treated titanium and zirconia surfaces (Figure 1A,C, Table 1). Specifically, after 2 and 24 h, viability of murine osteoblast-like cells (MT3C3) in all UV-light treated groups (1, 3, 6, 9, 12, 16 min) was significantly higher than the control group. Viability after 12 min of UV light treatment was higher than after other UV-light treatment times (Table 2).

Viability of MT3C3 cells was significantly higher after NTP treatment in all groups compared to controls after either 2 or 24 h of incubation (Figure 1B,D and Table 1). Viability of cells in 1 min oxygen NTP treated groups was highest among all groups for titanium after 2 and 24 h of incubation. Viability of cells in 1 min was significantly higher than that in 9 and 16 min treated groups for zirconia after 2 h (Figure 1B, Table 3, *p* < 0.05), and 16 min treated group for zirconia after 24 h (Figure 1D, Table 3, *p* < 0.05). 

### 2.2. Quantification of mRNAs

**Figure 2:**
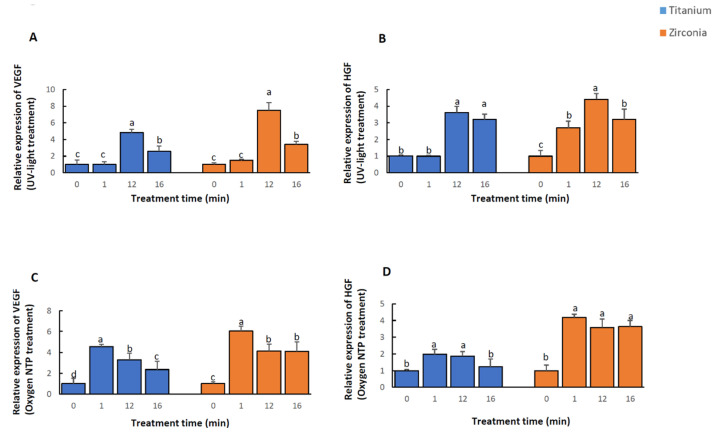
The relative gene vascular endothelial growth factor (VEGF) and hepatocyte growth factor (HGF) expressions of MC3T3-E1 after 24 h of culture following time-course surface treatment of titanium and zirconia with UV-light (**A**,**B**) and oxygen NTP (**C**,**D**). Each letter on top of each bar indicates the order of relative gene expression, where the letter a indicates the test or control group with the highest relative gene expression. The same letter indicates no statistically significant difference in relative gene expression between the groups. All of the statistical significance values were declared as *p* < 0.05.

The results are shown in Figure 2. The quantification of mRNA for each gene expression was analyzed and the relative expressions of VEGF after 12 and 16 min UV-light treated surfaces of both titanium and zirconia were significantly higher than those on the control group (Figure 2A, *p* < 0.05) which was also the case in HGF gene expression. Additionally, the relative expression of HGF was significantly higher on 1 min UV-light treated zirconia surfaces compared to controls (Figure 2B, *p* < 0.05). Generally, VEGF and HGF relative gene expression was significantly higher after 12 min of UV-light treatment compared to both 1 min and 16 min of UV-light treatment on titanium and zirconia samples (Figure 2A, *p* < 0.05). 

Unlike UV-light treatment, the mRNA relative expression of VEGF for all NTP treated groups was significantly higher compared to controls (Figure 2C, *p* < 0.05). This was the basically same case for HGF relative expression on oxygen NTP treated surfaces of zirconia. Except after 16 min of NTP treatment on titanium, the relative expression of HGF in other NTP treated groups was significant compared to controls (Figure 2D, *p* < 0.05). For all NTP treated titanium and zirconia groups, VEGF relative expression was significantly higher in 1 min treated groups compared to 12 min and 16 min treated groups. Additionally, relative expression of VEGF on 12 min treated titanium surfaces was significantly higher compared to 16 min of treatment (Figure 2C, *p* < 0.05). HGF relative expression in 1 min and 12 min NTP treated titanium groups was significantly higher compared to the 16 min treated group, while there was no statistically significant difference between 1, 12 and 16-min NTP treated zirconia groups (Figure 2D, *p* < 0.05).

### 2.3. Cell Attachment and Morphology

**Figure 3:**
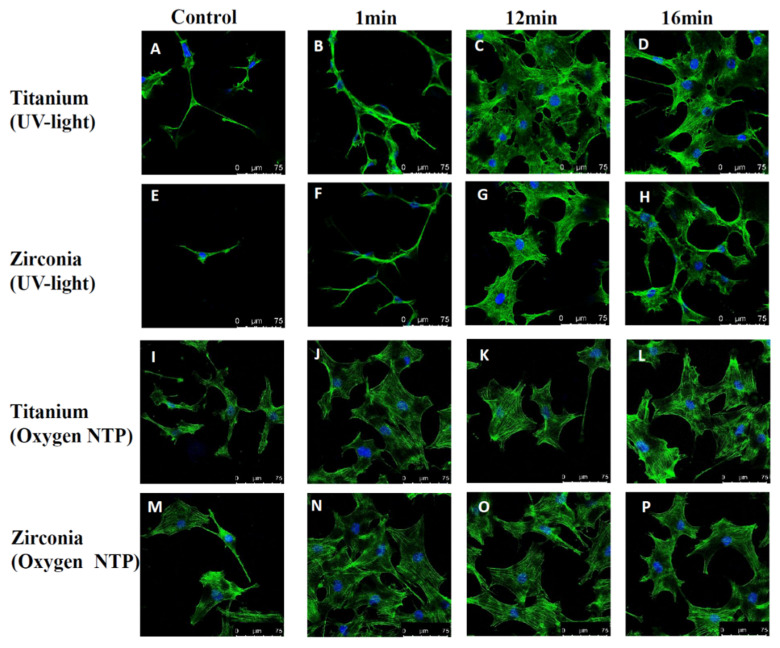
Representative examples of cytoskeleton stained with phalloidin after 24 h of incubation on controls (**A**,**E**,**I**,**M**), 1, 12, 16 min UV-light treated (**B**–**D**, **F**–**H**) and 1, 12, 16 min oxygen plasma treated (**J**–**L**,**N**–**P**) surfaces of titanium and zirconia using confocal microscopy.

Figure 3 shows the cell attachment and morphology of MC3T3-E1 cells after 24 h of culture on UV-light treated, NTP treated and control titanium and zirconia surfaces after 1 min, 12 min and 16 min of treatment. 

Cells on 12 min and 16 min UV-light treated and all NTP treated surfaces of titanium and zirconia showed regular morphology and size and a widely spread cytoskeleton. Additionally, cells on NTP treated titanium and zirconia surfaces showed a higher cell density after 1 min of surface treatment compared to 12 min and 16 min of treatment. Cells in control groups and cells on 1 min UV-light treated titanium and zirconia groups showed some morphologic irregularities, e.g., a thinner cytoskeleton and a less-stretched appearance. 

## 3. Discussion

In this study, the effects of different UV light treatment and non-thermal oxygen plasma treatment periods on viability, cell attachment and mRNA expression of VEGF and HGF in murine osteoblast-like cells on titanium and zirconia samples were investigated. To the authors’ knowledge, this is the first study to investigate the influence of different treatment durations of both methods. 

The results showed that the effect of UV-light and NTP treatment on the growth of MC3T3-E1 did not increase with a prolonged treatment duration. The viability of MC3T3-E1 in UV light treated groups was significantly higher after 12 min and 16 min of treatment and, generally, relative expression of VEGF and HGF reached the highest levels after 12 min of surface treatment. The viability of cells as well as relative expression of VEGF after only 1 min of non-thermal oxygen plasma treatment was significantly higher compared to any longer treatment durations. Cells on 12 min and 16 min UV-light treated titanium and zirconia surfaces had a widely spread cytoskeleton. However, cells on titanium and zirconia surfaces following a 1 min non-thermal oxygen plasma treatment showed no decrease in cytoskeleton and even had a higher cell density compared to longer treated surfaces. In this study, 12 min UV-light and 1 min non-thermal oxygen plasma treatments were superior treatment durations in improving MC3T3 cell survival and attachment. 

VEGF is believed to play a critical role in wound healing and bone repair [22,23,24]. An increasing number of studies report that VEGF may play a significant role in regulating osteoblast activity [24,25,26]. Studies by Deckers and Hah et al. found that VEGF produced by osteoblast-like cells can act as an autocrine growth factor and may also stimulate endothelial cells to secrete cytokines and growth factors for enhancing osteoblastic differentiation [25,26]. HGF is a multifunctional growth factor with a major role in tissue morphogenesis and repair [27,28]. HGF produced by osteoblasts (including MC3T3) has been considered as a regulator of skeletogenesis and bone remodeling via stimulating the proliferation and alkaline phosphatase activity in osteoblasts and promoting osteoblast differentiation [29,30,31]. Studies showed that HGF may be an excellent candidate for implant coating [32]. Therefore, in the present study, the mRNA expression of VEGF and HGF was included to evaluate the effect of UV irradiation and oxygen NTP treatment of different durations on the function of MC3T3. The results of the present study showed that the highest levels of VEGF expression on titanium and zirconia samples, as well as the highest levels of HGF expression on titanium samples, were reached after 12 min of UV-light and 1 min of non-thermal oxygen plasma treatment, suggesting that these treatment times would be most effective considering the machines and settings that were used. 

Regarding practicability, a shorter processing time may represent greater feasibility. Compared to 12 min UV light treatment, oxygen non-thermal plasma treatment may achieve the described effects within a shorter time, which may be more easily integrated into clinical routines. One of the possible reasons for this is that oxygen plasma may accelerate the oxygen saturation of the treated surfaces, as well as the formation of ROS, improving the hydrophilicity and cytocompatibility of these treated materials surfaces [11,12,16]. However, the underlying mechanisms still need further exploration. 

Cellular morphology is regarded as a critical parameter in the assessment of cellular activity due to the observation that the formation of an actin cytoskeleton with filopodia projections of stretched cells is mostly related to superior activity of cells [33]. Spread out cells can form adhesion molecules like an actin cytoskeleton, that is considered to be linked with cell attachment which plays an important role in proliferation and differentiation [34]. The results of cellular morphology were related to the gene expression analysis, since the results showed that cells on 12 min and 16 min UV-light treated surfaces of titanium and zirconia presented a widely spread cytoskeleton. Additionally, NTP treated surfaces of titanium and zirconia with a shorter time duration (1 min) displayed equivalent cell spread and even presented a higher density compared to 12 min and 16 min treated surfaces. However, cells in any control group and after 1 min UV light treatment on titanium and zirconia exhibited some morphologic irregularities, with a thin cytoskeleton and a less-spread appearance. Hence, 12 min UV-light and 1 min non-thermal oxygen plasma treatment resulted in a better pre-osteoblast function in this study. 

NTP is gaining more and more attention in medicine and biology. NTP can form hydroxyl groups on the surfaces of biomaterials by inducing a one-electron oxidation of atmospheric water leading to a hydrogen ion and a hydroxyl radical [35]. Comparable to UV light, hydroxyl groups can increase hydrophilicity, as well as chemical interactions between cells and surfaces of biomaterials [20]. Up to now, various plasma sources have also been used to sterilize implants and biological surfaces by the destruction of microorganisms and removal of biofilms [13,14,15]. After sterilization, the charged particles form free radicals which combine to form water and oxygen. When applied, free radicals evaporate, spread, and may successfully kill bacteria, viruses and fungi on all reached surfaces [36]. Plasma sources can also generate ROS, such as ozone, atomic oxygen, superoxide, peroxide radicals and hydroxyl radicals, visible and infrared radiation, neutral molecules, ions, electrons, and exited atoms [37]. It was reported that NTP may enhance the attachment and proliferation of stem cells without affecting their stem cell properties due to the generation of ROS [16]. A previously conducted study revealed that 12 min non-thermal oxygen plasma treatment significantly increased the cellular activity of human gingival fibroblasts and murine fibroblasts on 4 week old titanium and zirconia surfaces [21]. The results of the present study show that after a significant shorter treatment time of only 1 min, non-thermal oxygen plasma may even have increased application effects compared to longer processing durations—which may be due to the different degree of physico-chemical changes—on oxygen plasma treated surfaces. Although the underlying mechanism still needs further exploration, this also provides the possibility for a more convenient clinical use.

UV photofunctionalization has been reported as a method to increase hydrophilicity in order to improve cell attachment and bone formation on treated material surfaces [38,39,40,41]. Lee et al. treated hydroxyapatite grafting materials containing TiO2 with UV light (UV radiation with a peak at 253.7 nm, power: 8 W) in a dark room for 1 min and found that UV irradiation increased the extent of new bone formation in rabbit calvarial bone [42]. However, Jimbo et al. found that titanium implants that were treated by UV light for 24 h showed a significantly increased bone-to-implant contact after 2 and 6 weeks of healing in rabbit tibiae compared to controls [41]. In previous studies, results have shown that wettability and oxygen content of titanium and zirconia surfaces were significantly increased and the carbon content significantly decreased after 12 min of UV light treatment, which may lead to improved implant surface conditions after long-time storage in customary packages [12,19,20]. Although clinical practicability may increase with the reduction in processing time, an appropriate UV irradiation treatment time was still unclear. The present study revealed that a 12 min UV-light treatment might be optimal in a 1 to 16 min interval.

UV irradiation, as well as non-thermal oxygen plasma treatment, are promising methods to improve the biocompatibility of dental implant materials. They can easily be integrated into the routine of a dental practice due to the manageable size of the required devices and practicable processing times. This study evaluated different processing times of UV light and NTP on MC3T3 that were seeded on titanium or zirconia samples and indicated that the effects did not necessarily increase with a prolongation of treatment time. However, the underlying mechanism of this phenomenon still needs further investigation. Effects may change with different conditions of equipment like intensity of UV-light, generator frequency of the NTP reactor and flow rate of gas. In addition, it is astonishing that the ceramic material demonstrates such a different response as compared to titanium surfaces. Other studies have also suggested that bulk material properties may play a role in cell behavior. The results of this study were able to confirm these suggestions.

The limitation of the current study is that it is only an in vitro characterization. The clinical implications of the determined effects have to be evaluated in further studies. Additionally, using a single cell line is another limitation of the study since cell lines may not adequately represent primary cells’ reactions. Therefore, further and in-depth research, for example analysis of different treated surfaces, the exploration of osteo-differentiation and in vivo studies are needed to rank and classify the results of this in vitro study into bigger contexts.

## 4. Materials and Methods

### 4.1. Sample Preparation 

Specimen of 15 mm diameter and 1.5 mm thickness were made from pure grade 4 titanium (Camlog, Basel, Switzerland). Zirconia disks were made from tetragonal zirconia polycrystal (ZrO_2_ 95%, Y2O3 5%, 15 mm in diameter, 1.5 mm in thickness; Camlog, Basel, Switzerland). Surfaces of all titanium and zirconia samples were sterilized and stored in customary packages for at least 4 weeks.

### 4.2. UV-Light and NTP Treatment

Surfaces of titanium and zirconia were treated by UV light or non-thermal oxygen plasma with increasing duration (0, 1, 3, 6, 9, 12 and 16 min). All samples were randomly divided into one group of non-treated samples (0 min, control group) and six experimental groups according to treatment duration. UV light was generated using an UV light oven with an intensity of 0.15 mW/cm^2^ (λ = 253.7 nm). Oxygen plasma was produced using an NTP reactor (generator frequency 100 kHz, input power 24 W, system pressure 1mbar, gas flow rate 1.25 sccm, and gas purity >99.5%, Diener Electronic GmbH, Ebhausen, Germany). 

### 4.3. Cell Culture

Murine osteoblast-like cells MC3T3-E1 (C57BL/6, Sigma-Aldrich^®^, Munich, Germany) were used for all experiments. Cells were cultured in α-modified minimum essential medium with nucleosides (MEM α Gibco™, Invitrogen™, Paisley, UK) supplemented with 10% fetal bovine serum (FBS Gibco™, Invitrogen™, Paisley, UK) and 1% penicillin/streptomycin (P/S Gibco™, Invitrogen™, Paisley, UK). Cells were incubated in a humified atmosphere of 95% air and 5% CO_2_ at 37 °C. They were detached at 80% confluence using 0.05% trypsin with ethylenediaminetetraacetic acid (Gibco™, Invitrogen™, Paisley, UK) and counted in a hemocytometer (Hecht Assistant, Sondheim vor der Rhon, Germany). In order to access cell attachment and morphology, cells were seeded onto the treated or non-treated disks at a density of 0.5 × 10^5^/cm^2^. Cell viability was assessed using a density of cells of 1 × 10^5^/cm^2^.

### 4.4. Viability Assay 

After 2 and 24 h of incubation, the viability of cells was assessed using CellTiter 96^®^ Aqueous Non-Radioactive Cell Proliferation Assay Kits (MTS assay, Promega, Madison, WI, USA). Briefly, a one-fifth volume of MTS solution was added to each well and the plates were incubated for 1–4 h at 37 °C in a humidified, 5% CO_2_ atmosphere. The absorbance was measured using a microplate reader at a wavelength of 490 nm.

### 4.5. Gene Expression Analysis

The effects of UV light and non-thermal oxygen plasma on the expression of various messenger ribonucleic acids (mRNAs) were assessed using real-time reverse transcription polymerase chain reaction (qRT-PCR) analysis. Total RNA from cells of each experimental and control group was isolated using the TRIzol reagent (Invitrogen, Grand Island, NY, USA) after 24 h of cell culture. Complementary deoxyribonucleic acid (cDNA) was synthesized using random primers and standard protocols which was followed by performing qRT-PCR using a SsoAdvanced™ Universal Probes Supermix reagent (Bio-Rad, Benchmark, Hercules, CA, USA). mRNA of HGF and VEGF in each sample was measured in three replicates using dual-probe real-time PCR. One for the either of target mRNA (HGF or VEGF) and the other for mRNA of a reference housekeeping gene GAPDH. Cycle numbers at a defined threshold for target mRNA (Ct HGF or VEGF) and GAPDH (Ct GAPDH) were read and the difference between the two was calculated as ΔCt = Ct _HGF (or VEGF)_ − Ct _GAPDH_. Subsequently, relative copy number of HGF (or VEGF) mRNA to fictive 1000 copies of GAPDH-mRNA was calculated as 1000/2^ΔCt^. All values in experimental groups were normalized by the mean values of their corresponding control group.

### 4.6. Cell Attachment and Morphology

Confocal laser scanning microscopy (TCS SP8 X, Leica Microsystems, Wetzlar, Germany) was used to assess cell attachment and morphology using a 60-fold objective lens. Cell attachment in groups that were not treated or treated with UV-light or NTP after 1, 12 and 16 min was observed after 24 h of incubation. Cells were fixed by 4% paraformaldehyde for 30 min, and permeabilized with 0.1% Triton X-100/PBS (Gibco, Invitrogen, Paisley, UK) for 15 min at room temperature. After rinsing three times using PBS, F-actin filaments were stained using a fluorescent dye (biotinylated phalloidin, Alexa Fluor 488 green, 1:1000; Thermo Fisher Scientific, Waltham, MA, USA) and incubated for 60 min at room temperature. After that, samples were washed with PBS for three times and dried in normal air. Antifade Mountant (Fluoromount-G, Southern Biotech, AL, USA) was used to fix all samples on glass-bottom dishes (WillCo-Dish, Amsterdam, The Netherlands) and stored in the dark at 4 °C.

### 4.7. Statistical Analysis

Statistical analysis was performed using SPSS 21 (IBM, Armonk, NY, USA). Normality of viability values and gene expression was assessed using the skewness–kurtosis method. Afterwards, data were analyzed using a one-way analysis of variance (ANOVA) in cases of normal distribution. For skewed data, non-parametric Kruskal–Wallis tests were used. For all results, statistical significance was set at *p* < 0.05.

## 5. Conclusions

As regards the limitations of this in vitro study, the results indicated that 12 min of UV-light treatment and 1 min of non-thermal oxygen plasma surface treatment on titanium and zirconia may be appropriate in terms of increasing the viability, mRNA expression of growth factors and cellular attachment of osteoblast-like cells.

## Figures and Tables

**Table 1:** Viability of cells after 2 and 24 h of incubation.

		Control	1 min	3 min	6 min	9 min	12 min	16 min
Titanium 2 h	UV	0.312 (0.015)	0.376 ^ab^ (0.018)	0.381 ^ab^ (0.018)	0.362 ^ab^ (0.018)	0.372 ^ab^ (0.013)	0.409 ^a^ (0.008)	0.390 ^a^ (0.016)
	Oxygen NTP	0.312 (0.015)	0.403 ^a^ (0.011)	0.341 ^ac^ (0.015)	0.344 ^ac^ (0.019)	0.361 ^ac^ (0.019)	0.344 ^ac^ (0.014)	0.341 ^ac^ (0.010)
Zirconia 2 h	UV	0.189 (0.010)	0.269 ^ab^ (0.012)	0.269 ^ab^ (0.009)	0.276 ^a^ (0.013)	0.280 ^a^ (0.016)	0.290 ^a^ (0.009)	0.281 ^a^ (0.002)
	Oxygen NTP	0.189 (0.010)	0.271 ^a^ (0.017)	0.258 ^a^ (0.052)	0.251 ^a^ (0.022)	0.223 ^ac^ (0.020)	0.248 ^a^ (0.017)	0.232 ^ac^ (0.014)
Titanium 24 h	UV	0.336 (0.011)	0.359 ^ab^ (0.007)	0.401 ^ab^ (0.014)	0.389 ^ab^ (0.005)	0.407 ^ab^ (0.010)	0.493 ^a^ (0.017)	0.473 ^a^ (0.019)
	Oxygen NTP	0.336 (0.011)	0.460 ^a^ (0.016)	0.409 ^ac^ (0.010)	0.414 ^ac^ (0.005)	0.411 ^ac^ (0.015)	0.424 ^ac^ (0.016)	0.393 ^ac^ (0.004)
Zirconia 24 h	UV	0.251 (0.018)	0.269 ^b^ (0.018)	0.329 ^ab^ (0.008)	0.342 ^ab^ (0.012)	0.344 ^ab^ (0.019)	0.375 ^a^ (0.008)	0.354 ^a^ (0.011)
	Oxygen NTP	0.251 (0.018)	0.331 ^a^ (0.023)	0.325 ^a^ (0.030)	0.315 ^a^ (0.011)	0.312 ^a^ (0.007)	0.308 ^a^ (0.002)	0.296 ^ac^ (0.029)

UV: ultraviolet, NTP: non-thermal plasma; Values are given as mean (SD); ^a^ compared to control; ^b^ compared to 12-min treatment; ^c^ compared to 1-min treatment.

**Table 2:** Statistical results of one-way analysis of variance test in 12 min UV-light treated groups.

	Titanium 2 h	Zirconia 2 h	Titanium 24 h	Zirconia 24 h
P_covs12(min)_	<0.001	<0.001	<0.001	<0.001
P_1vs12(min)_	0.019	0.029	<0.001	<0.001
P_3vs12(min)_	0.041	0.033	<0.001	0.001
P_6vs12(min)_	0.002	0.124	<0.001	0.011
P_9vs12(min)_	0.011	0.288	<0.001	0.015
P_16vs12(min)_	0.144	0.321	0.077	0.082

**Table 3:** Statistical results of one-way analysis of variance test in 1 min oxygen nonthermal plasma treated groups.

	Titanium 2 h	Zirconia 2 h	Titanium 24 h	Zirconia 24 h
P_covs1(min)_	<0.001	<0.001	<0.001	<0.001
P_3vs1(min)_	<0.001	0.403	<0.001	0.685
P_6vs1(min)_	<0.001	0.220	0.001	0.327
P_9vs1(min)_	0.004	0.007	<0.001	0.242
P_12vs1(min)_	<0.001	0.161	0.004	0.163
P_16vs1(min)_	<0.001	0.025	<0.001	0.046

## Abbreviations

NTP	Non-thermal plasma
UV	Ultraviolet
ROS/RNS	reactive oxygen/nitrogen species
VEGF	vascular endothelial growth factor
HGF	hepatocyte growth factor
